# Viability of *Baylisascaris procyonis* Eggs

**DOI:** 10.3201/eid1707.101774

**Published:** 2011-07

**Authors:** Shira C. Shafir, Frank J. Sorvillo, Teresa Sorvillo, Mark L. Eberhard

**Affiliations:** Author affiliations: University of California Los Angeles School of Public Health, Los Angeles, California, USA (S.C. Shafir, F.J. Sorvillo, T. Sorvillo);; Los Angeles County Department of Public Health, Los Angeles (F.J. Sorvillo);; Centers for Disease Control and Prevention, Atlanta, Georgia, USA (M.L. Eberhard);; Trident University International, Cypress, California, USA (F.J. Sorvillo)

**Keywords:** Baylisascaris procyonis, eggs, viability, helminth, infection, neurologic disease, parasites, California, dispatch

## Abstract

Infection with *Baylisascaris procyonis* roundworms is rare but often fatal and typically affects children. We attempted to determine parameters of viability and methods of inactivating the eggs of these roundworms. Loss of viability resulted when eggs were heated to 62°C or desiccated for 7 months but not when frozen at –15°C for 6 months.

*Baylisascaris procyonis*, the common intestinal roundworm of raccoons, has increasingly been recognized as a source of severe, often fatal, neurologic disease in humans, particularly children (*1**,**2*). Although this devastating disease is rare, lack of effective treatment and the widespread distribution of raccoons in close association with humans make baylisascariasis a disease that seriously affects public health (*3*). Raccoons infected with *B. procyonis* roundworms can shed millions of eggs in their feces daily (*4*). Given the habit of raccoons to defecate in and around houses, information about optimal methods to inactivate *B. procyonis* eggs are critical for the control of this disease. However little information is available about survival of eggs and effective disinfection techniques. This study expands on our previous work by providing additional data on thermal death point and determining the impact of desiccation and freezing on the viability of *B. procyonis* eggs to provide additional information for risk assessments of contamination and guide attempts at environmental decontamination (*5*).

## The Study

Worms were harvested from the intestines of infected raccoons from Goleta, California, USA. Eggs were removed from adult female worms and allowed to embryonate in sterile saline at room temperature for 3 weeks.

**Figure 1:**
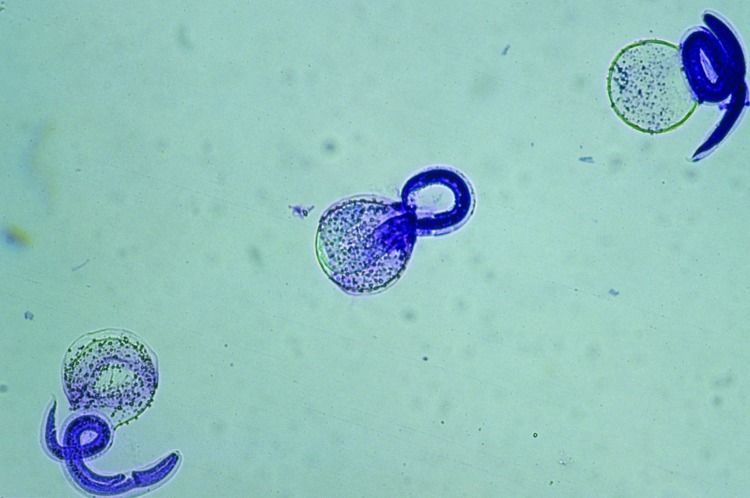
Viable *Baylisacaris procyonis* larvae demonstrating intact membrane and impermeability to methylene blue. Original magnification ×40.

**Figure 2:**
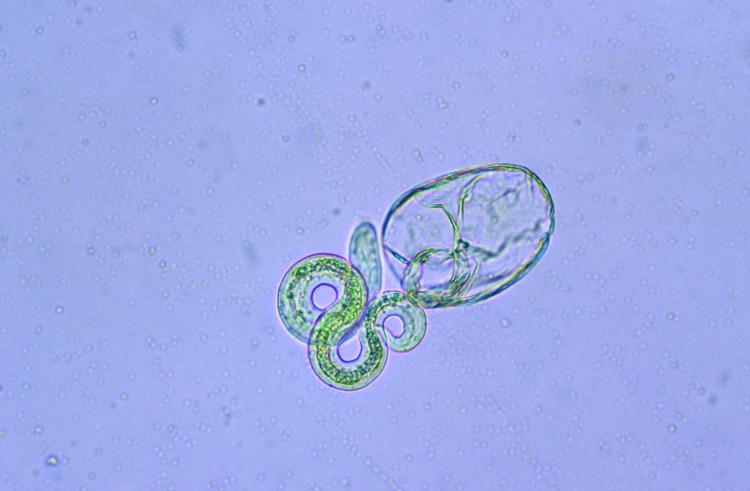
Hatched nonviable *Baylisascaris procyonis* larvae demonstrating uptake of methylene blue. Original magnification ×40.

To determine the thermal death point, 150 μL each of embryonated eggs, at a concentration of 100 eggs per μL were added to six 1-mL polypropylene tubes of sterile water. The 6 tubes were then added to a water bath at 35°C and allowed to sit for 10 min to equilibrate. Temperature of the water bath was increased at a rate of ≈5°C every 7 min, and 1 tube was removed at 5° increments from 37°C to 62°C. Eggs were then examined by light microscopy to determine viability as judged by larval motility. A minimum of 50 eggs were examined per assay, and larval motility was judged as +/–. The experiment was repeated using a more objective assessment of viability through examination of hatched larvae. Inactivation was measured by using a viability dye (methylene blue) exclusion method where uptake of dye indicates cell death and inactivation. After the eggs were removed from the heat, the mammilated layer was removed through exposure to undiluted household bleach (6% sodium hypochlorite) to promote subsequent emerging of larvae, then washed 5 times in 0.85% saline for 1 min at 600 × *g*. Hatching was achieved by the glass bead method (*6**,**7*). Hatched larvae were then removed and mixed 1:1 with a 1:10,000 dilution of methylene blue. Viable larvae remained motile and remained unstained (Figure 1), whereas nonviable larvae absorbed the methylene blue stain (Figure 2) (*8*).

The experiment was repeated by adding the heated water, in 5° increments from 37°C to 62°C, directly into the tube containing the eggs. Eggs were exposed to the water for <1 min. Eggs were then processed as described and examined by light microscopy. All experiments were replicated.

To determine the ability of eggs to withstand freezing temperatures, 150 μL each of embryonated eggs at a concentration of 100 eggs per μL were added to ten 1-mL polypropylene tubes of sterile water. The tubes of eggs were exposed to an environment of –15°C. Every 30 days, 1 tube was removed and thawed at room temperature. Viability was evaluated by using both motility and methylene dye exclusion as described above. After examination, tubes were refrozen. Each month, in addition to the evaluation of a fresh tube, tubes from the previous months were reevaluated to examine the effects of freeze–thaw. A total of 5 freeze–thaw cycles were evaluated.

To assess survival of eggs to total desiccation, 150 μL each of embryonated eggs, at a concentration of 100 eggs per μL, were added to ten 1-mL wells of sterile water in a microtiter plate. The plates were stored at room temperature and allowed to dehydrate to complete desiccation over a 1-week period. Monthly, 1 mL of sterile water was added and evaluated for eggs and assessed for viability. Assessments were made until complete inactivation occurred.

All larvae remained viable to 47°C; >90% of the larvae were viable at 52°C, and 50% were viable at 57°C. Larve were completely inactivated at 62°C (Table). When the heated water was added directly to the eggs, all larvae remained viable through 42°C, and most larvae were viable at 47°C and 52°C. Complete inactivation occurred at 57°C (Table).

**Table:** Viability of *Baylisacaris procyonis* eggs in sterile water when exposed to 2 different heating conditions

Temperature water slowly heated to, °C	% Viable	Temperature of hot water added, °C	% Viable
37°C	100	37	100
42°C	100	42	100
47°C	100	47	98
52°C	94	52	92
57°C	50	57	0
62°C	0	62	0

Eggs survived complete desiccation for at least 6 months at room temperature. Total loss of viability was observed after 7 months of desiccation. Eggs frozen at –15°C for 6 months demonstrated no reduction in viability. Freeze–thaw, including exposure to 5 cycles, did not affect survival. Although we did not specifically design experiments to assess the effect of chlorine on inactivation of *B. procyonis* eggs, exposure to undiluted household bleach for 90 min to remove the mammillated layer did not affect viability.

## Conclusions

These findings indicate that *B. procyonis* eggs have a low thermal death point at <62°C, similar to that reported for other ascarids (*5**,**9*). Moreover, inactivation was achieved at relatively short exposure times. However, the eggs are highly resistant to desiccation. Additionally, extended freezing or freeze–thaw exposure is unlikely to have affect viability, similar to *Parascaris equorum* (*10*). Exposure to undiluted bleach had no observable effect on survival.

In North America, the prevalence of *B. procyonis* is high, and infection is possible wherever raccoons are found (*11**–**14*). Given the close association of raccoons with human populations and the serious nature of infection, identification of the parameters of viability of *B. procyonis* larvae has public health implications (*15*). Potential for human infection can be mitigated by decontamination of areas known to be contaminated with raccoon feces. Such contamination of yards, pools, and homes is commonly reported. Hyperchlorination of contaminated swimming pool water is unlikely to be effective. However, current recommendations are to completely drain water and refill pools or to allow complete filtration and turnover of water and to discard and replace the filter material. Given the low thermal death point of *B. procyonis* eggs, the use of readily available steam-producing devices may be of value in disinfecting contaminated areas. Moreover, because freezing and freeze–thaw do not affect viability, contamination in areas that have cold winters represents potential risk. Because eggs can survive periods of prolonged desiccation, areas contaminated with raccoon feces that have dried cannot be considered safe. However, the recognition of complete inactivation of eggs at relatively low temperatures provides guidance in circumstances where contamination with *B. procyonis* eggs requires disinfection efforts and indicates that approaches well short of incineration or boiling will be effective. Furthermore, these results suggest that temperatures achievable in point-of-use hot water heaters (household units) can deactivate infectious *B. procyonis* eggs and provide an option for maintaining safe drinking water during a possible bioterrorism event or a boil water advisory. Further studies are needed to determine the effectiveness of heat and other possible disinfection methods on inactivation of eggs in natural circumstances, such as contaminated play areas and soil.

## References

[1] Sorvillo F, Ash LR, Berlin OG, Morse SA. *Baylisascaris procyonis*: an emerging helminthic zoonosis. Emerg Infect Dis. 2002;8:355–9. 10.3201/eid0804.010273 11971766 PMC2730233

[2] Shafir SC, Wise ME, Sorvillo FJ, Ash LR. Central nervous system and eye manifestations of infection with *Baylisascaris procyonis.* Curr Infect Dis Rep. 2006;8:307–13. 10.1007/s11908-006-0076-7 16822375

[3] Wise ME, Sorvillo FJ, Shafir SC, Ash LR, Berlin OG. Severe and fatal central nervous system disease in humans caused by *Baylisascaris procyonis*, the common roundworm of raccoons: a review of current literature. Microbes Infect. 2005;7:317–23. 10.1016/j.micinf.2004.12.005 15715975

[4] Snyder DE. Contaminative potential, egg prevalence, and intensity of *Baylisascaris procyonis*–infected raccoons (*Procyon lotor*) from Illinois, with a comparison to worm intensity. Proc Helminthiol Soc Wash. 1987;54:141–5.

[5] Shafir SC, Wang W, Sorvillo FJ, Wise ME, Moore L, Sorvilo T, Thermal death point of *Baylisascaris procyonis* eggs. Emerg Infect Dis. 2007;13:172–3. 10.3201/eid1301.060966 17370540 PMC2725829

[6] Sheppard CH, Kazacos KR. Susceptibility of *Peromyscus leucopus* and *Mus musculus* to infection with *Baylisascaris procyonis.* J Parasitol. 1997;83:1104–11. 10.2307/3284370 9406787

[7] Urban JF Jr, Douvres FW. In vitro development of *Ascaris suum* from third- to fourth-stage larvae and detection of metabolic antigens in multi-well culture systems. J Parasitol. 1981;67:800–6. 10.2307/3280703 7328453

[8] Boyce WM, Branstetter BA, Kazacos KR. Comparative analysis of larval excretory–secretory antigens of *Baylisascaris procyonis, Toxocara canis* and *Ascaris suum* by Western blotting and enzyme immunoassay. Int J Parasitol. 1988;18:109–13. 10.1016/0020-7519(88)90044-6 3366527

[9] Feachem R, Bradley DJ, Garelick H, Mara DD. *Ascaris* and ascariasis. In: Sanitation and disease: health aspects of excreta and wastewater management. Washington: World Bank: 1983. p. 391.

[10] Koudela B, Bodecek S. Effects of low and high temperatures on viability of *Parascaris equorum* eggs suspended in water. Vet Parasitol. 2006;142:123–8. 10.1016/j.vetpar.2006.05.031 16876952

[11] Kazacos KR. *Baylisascaris procyonis* and related species. In: Samuels WM, Pybus MJ, Kocans AA, editors. Parasitic diseases of wild mammals. 2nd ed. Ames (IA): Iowa State University Press; 2001. p. 301–41.

[12] Yeitz JL, Gillin CM, Bildfell RJ, Debess EE. Prevalence of *Baylisascaris procyonis* in raccoons (*Procyon lotor*) in Portland, Oregon, USA. J Wildl Dis. 2009;45:14–8. 19204331 10.7589/0090-3558-45.1.14

[13] Page LK, Gehrt SD, Robinson NP. Land-use effects on prevalence of raccoon roundworm (*Baylisascaris procyonis*). J Wildl Dis. 2008;44:594–9. 18689644 10.7589/0090-3558-44.3.594

[14] Yabsley MJ, Blizzard EL, Beck MF, Harsch S. Geographic expansion of *Baylisascaris procyonis* roundworms, Florida, USA [letter] [**PMID: 29029553**]. Emerg Infect Dis. 2010;16:1803–4. 21029553 10.3201/eid1611.100549 PMC3294519

[15] Page LK, Anchor C, Luy E, Kron S, Larson G, Madsen L, Backyard raccoon latrines and risk for *Baylisascaris procyonis* transmission to humans. Emerg Infect Dis. 2009;15:1530–1. 10.3201/eid1509.090128 19788835 PMC2819851

